# Supported quantum clusters of silver as enhanced catalysts for reduction

**DOI:** 10.1186/1556-276X-6-123

**Published:** 2011-02-08

**Authors:** Annamalai Leelavathi, Thumu Udaya Bhaskara Rao, Thalappil Pradeep

**Affiliations:** 1 DST Unit of Nanoscience (DST UNS), Department of Chemistry, Indian Institute of Technology Madras, Chennai 600036, India

## Abstract

Quantum clusters (QCs) of silver such as Ag_7_(H_2_MSA)_7_, Ag_8_(H_2_MSA)_8 _(H_2_MSA, mercaptosuccinic acid) were synthesized by the interfacial etching of Ag nanoparticle precursors and were loaded on metal oxide supports to prepare active catalysts. The supported clusters were characterized using high resolution transmission electron microscopy, scanning electron microscopy, X-ray photoelectron spectroscopy, and laser desorption ionization mass spectrometry. We used the conversion of nitro group to amino group as a model reaction to study the catalytic reduction activity of the QCs. Various aromatic nitro compounds, namely, 3-nitrophenol (3-np), 4-nitrophenol (4-np), 3-nitroaniline (3-na), and 4-nitroaniline (4-na) were used as substrates. Products were confirmed using UV-visible spectroscopy and electrospray ionization mass spectrometry. The supported QCs remained active and were reused several times after separation. The rate constant suggested that the reaction followed pseudo-first-order kinetics. The turn-over frequency was 1.87 s^-1 ^per cluster for the reduction of 4-np at 35°C. Among the substrates investigated, the kinetics followed the order, SiO_2 _> TiO_2 _> Fe_2_O_3 _> Al_2_O_3_.

## Introduction

Monolayer-protected quantum clusters (QCs) composed of a few atoms show unique properties due to their novel atomic and electronic structure. Their discrete electronic states produce well-defined luminescence in clusters, such as Au_25_, Au_23_, Au_22_, Au_8_, etc. [1-7]. They have attracted the attention of various fields such as sensors, biolabels, live cell-targeted imaging [4], single molecule electroluminescence [8], opto-electronics [9], and catalysis [10]. In the case of Au_25_, single crystal X-ray analysis has shown that it has an Au_13 _core protected with six [Au(SR)_2_] units in a core-shell-like pattern. The icosahedral Au_13 _core has 20 triangular faces. However, only 12 facets are face-capped by the exterior 12 Au atoms which keep eight facets open [1]. These "hole" sites may be useful as active sites [11] which may participate in catalytic processes. Although the single crystal structures of the most of the clusters are yet to be solved, it is expected that many of them contain active sites. In this study, electron transfer properties of Ag_7 _and Ag_8 _clusters were explored using a simple reduction reaction, namely, the conversion of nitro to amino group, in different substrates. Catalysis using noble metals has caused great excitement after the initial report of Haruta [12]. It is known that reduction potential of silver nanoparticles change with size and shifts to more negative values with decrease in size, which are in good agreement with previous predictions [13]. QCs have very high negative reduction potential in comparison to bulk, and this makes them useful for the catalysis of electron transfer reactions. Silver is less expensive than other noble metals, and it was reported as an efficient catalyst for several reactions. For example, silver on alumina is a promising catalyst for selective catalytic reduction of NO by hydrocarbons from automobile exhausts [14]. It has been reported that Ag nanoparticles on hydroxyapatite in the presence of water catalyze the selective oxidation of various phenylsilanes into phenylsilanols [15] and also that the same system was found to be a highly efficient catalyst for the selective hydration of nitriles to amides [16]. It has been reported that chloroanilines were produced in large scale by the hydrogenation of chloronitrobenzenes using Ag nanoparticles on SiO_2_, and the system also showed the size-dependent catalytic activity [17]. Formation of subsurface oxygen on the catalyst, enhanced in the case of supported silver clusters, plays an important role in CO oxidation [18]. Ag clusters supported on alumina are better active when compared to platinum supported on alumina for oxidant-free alcohol dehydrogenation to carbonyl compounds, and clusters less than 1 nm show structure-sensitive reactions [19]. It is reported that alumina is the best support for Ag, and it shows better selectivity for oxidation of ammonia to form nitrogen at low temperatures [20]. Silver nanoclusters on TiO_2 _enhance the reduction of bis (2-dipyridyl) disulfide to 2-mercaptopyridine in the presence of water [21]. It has been reported that the selectivity for hydrogenation of crotonaldehyde was very high in the presence of Ag catalyst below 3-nm diameter; the catalyst was also structure sensitive [22]. Shimizu et al. [23] reported that clusters of silver on alumina catalyze the cross-coupling reactions of alcohols, direct amide synthesis from alcohols and amines [24], and chemoselective reduction of a nitrostyrene with size-dependent catalytic activity [25]. The highest yield was obtained with particles of size ranging from 0.9 to 30 nm for the *N*-alkylation of anilines with benzyl alcohol for which silver shows high selectivity compared to other catalysts due to less stable metal-hydride bond formation [26]. Several reports are available for the reduction of nitro groups using nanoparticles [27-32]. Pal et al. reported the reduction of 4-nitrophenol (4-np) using silver nanoshells stabilized with cationic polystyrene beads [27] as well as silver deposited on silica gel [28]. It was reported that gold nanoparticles containing membranes reduce aromatic nitro compounds [29]. Ag and Au nanoparticles grown on calcium alginate gel beads are found to catalyze nitrophenol reduction [33].

In this article, we studied the catalysis of supported QCs of Ag_7,8 _using 4-np as the model system. Ag_7 _and Ag_8 _are new QCs prepared efficiently by the interfacial method [34]. Similar silver clusters are also made by other routes [35]. The reduction reaction occurs with a rate constant of 8.23 × 10^-3 ^at 35°C, and the TOF measured was 1.87 s^-1 ^per cluster. Performance of various supports has been evaluated.

## Experimental section

### Materials

All the following chemicals were commercially available and were used without further purification. Silver nitrate (AgNO_3_, 99%), mercaptosuccinic acid (MSA, 97%), methanol (GR grade), toluene (GR grade), and alumina were purchased from SRL Chemical Co. Ltd., India Mumbai. Trisodium citrate (Qualigens) Mumbai, India, titanium dioxide (Ranbaxy Fine Chemicals Limited) Mumbai, India, silica (Sisco Research Laboratories Private Limited) Mumbai, India, and iron oxide (Merck Specialties Private Limited) Mumbai, India were purchased from the mentioned laboratories. Sodium borohydride (NaBH_4_, 98%) was purchased from Sigma Aldrich. 4-np (C_6_H_5_NO_3_, 97%) and 4-aminophenol (C_6_H_7_NO, 98%) were purchased from Loba Chemicals. 3-np (C_6_H_5_NO_3, _97%), 3-na (C_6_H_6_N_2_O_2, _98%) and 4-na (C_6_H_6_N_2_O_2, _98%) were purchased from SD fine chemicals.

### Preparation of Ag@MSA nanoparticles

Mercaptosuccinic acid-capped silver nanoparticles were synthesized according to the reported method [36]. MSA (1795 mg) was dissolved in methanol (400 ml) and the mixture was kept under vigorous stirring in an ice bath. A solution of silver nitrate (340 mg) in 6.792 ml water was added. A freshly prepared aqueous solution of sodium borohydride (756.6 mg in 100 ml of water) was added drop by drop. The colorless solution changed to yellow, and further addition of NaBH_4 _changed it to brown. The solution was kept for half an hour for stirring. The particles were allowed to settle down in methanol, which were filtered and washed with methanol. The sample was again dispersed in methanol and centrifuged to remove excess thiols attached on the surface of the particles. The solvent was removed by rotavapor, in order to get a powder. These particles were freely dispersible in water. The UV-vis absorption spectrum shows a plasmon absorption around 390 nm for the as-prepared metallic Ag@MSA nanoparticles.

### Preparation of Ag QCs

Silver QCs were prepared by the interfacial etching method as per our earlier article [34]. In brief, 100 ml of toluene was added to the MSA solution (MSA 300 mg/100 ml of toluene/75 ml of water). This forms two phases, and the mixture was kept under vigorous stirring. To this, 100 mg of Ag@MSA nanoparticles in 25 ml of water was added (nanoparticles and MSA were in 1:3 ratio by mass). The etching process took place at the interface of the two phases (water/toluene). The color of the aqueous phase changed to yellow from dark brown. After 48 h of continuous stirring, it changed to orange. In the UV-vis absorption spectrum, the plasmon peak disappeared, which shows that no metallic behavior was retained in Ag QCs, and a new peak around 550 nm appeared due to intra-band transitions. The formed QCs were dissolved in the aqueous phase and were separated by freeze drying to obtain a powder. Mixture of dried QCs had an Ag_8_:Ag_7 _ratio of 80:20 [34]. Although they were separated in the earlier article [34], the mixture was used directly in this study in view of the quantities needed.

### Ag QCs supported on alumina

Alumina powder (60-325 mesh BSS) was added to aqueous Ag_8,7 _QC solutions, and the mixture was stirred for 5 min. Color of the alumina powder changed to orange, indicating that the QCs of Ag_7,8 _got loaded on alumina. The intensity of the color in the solution decreased, and finally, the solution became colorless. The amount of QCs in the solution was controlled to get various weight ratios of loading. The QC loaded materials were washed with water and dried in ambient air. Maximum loading corresponded to 0.1/1 g.

### Preparation of Ag@citrate nanoparticles

The Ag@citrate nanoparticles were prepared according to a previously published procedure [37]. They were loaded on alumina (10% loading), as in the case of QCs.

### Catalytic test

For the reduction reaction, 1 ml of freshly prepared ice-cold aqueous solution of NaBH_4 _(160 mM) was introduced to 1 ml of aqueous 4-np solution (7 mM), taken in a sample bottle. Next, Al_2_O_3_@Ag_7,8 _(10% loading, 50 mg) was added to the above solution mixture, and time-dependent absorption spectra were measured. From changes in the absorption of 4-nitrophenolate ion at 400 nm as a function of time, the rate constants were calculated. The product was identified by comparing with the spectrum of an authentic sample of 4-aminophenol (4-ap). The experiment was carried out at 15, 25, and 35°C.

### Instrumentation

UV-vis optical absorption spectra were recorded with a Perkin-Elmer Lambda 25 instrument. Fluorescence spectroscopy measurements were carried out using a HORIBA JOBIN VYON Nano Log instrument. XPS spectra were recorded using an Omicron ESCA Probe spectrometer with unmonochromatized Mg K_α _X-rays (*hυ *= 1253.6 eV). The samples were spotted as drop cast films on a sample stub. HRTEM of QCs coated on alumina was carried out using a JEOL 3010 instrument. The samples were cast on carbon-coated grids, and dried under ambient conditions. Scanning electron microscopy (SEM) and EDAX measurements were performed using a HITACHI S-4800 FESEM, and the samples were spotted on indium tin oxide (ITO) glass plates, followed by drying under ambient conditions. ESI-MS measurements were performed using a MDX Sciex 3200 Q-TRAP LC/MS/MS (Applied Biosystems) DST Unit of Nanoscience, IITM in which the spray and extraction are orthogonal to each other. Product formed was made to 10 ppm (1:1 ratio of water and methanol) and sprayed at 5 kV. LDI-MS studies were conducted using a Voyager DE PRO Biospectrometry Workstation of Applied Biosystems MALDI-TOF MS. A pulsed nitrogen laser of 337 nm was used for the studies.

## Results and discussion

**Figure 1:**
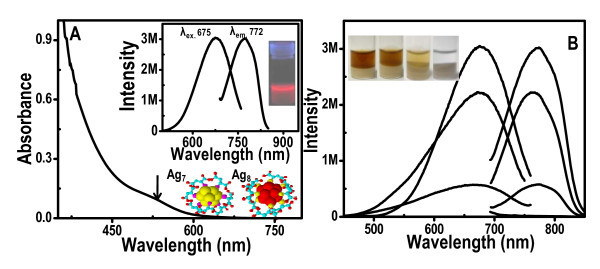
**UV-vis spectrum and luminescence spectra of the QC solution**. **(A) **UV-vis spectrum of Ag_7,8 _QCs. Inset of (a) shows the corresponding luminescence spectrum collected at 273 K along with a photograph of the red emitting crude cluster solution under UV-lamp at 273 K. **(b) **Decreasing intensity of luminescence spectra of the QC solution with increase in time, after alumina powders were added. Photograph in the inset shows the decrease in the intensity of the color of the solution with time as clusters are loaded on alumina (under white light illumination).

The as-synthesized QCs of Ag were characterized by optical absorption studies. The corresponding absorption spectra are shown in Figure 1a, and the peak around 550 nm is due to HOMO-LUMO transitions from the 4d valence band to the 5sp conduction band-derived states of QCs [34,38,39]. These were observed after 48 h of etching process [34] and the absence of nanoparticles was confirmed using high resolution transmission electron microscopy of the etched materials. This confirmed that the peak around 550 nm was due to clusters. Inset of Figure 1a shows the photoluminescence spectrum of Ag_7,8 _QCs having an excitation at 675 nm and emission at 772 nm; these data were collected at 273 K, and the photograph in the inset corresponds to red emission from the as-prepared crude mixture of the QC solutions under UV light irradiation. Figure 1b shows that the emission intensity of the solution decreased with the addition of alumina powder. As QCs got coated on alumina, the concentration of QCs in the solution decreases, and finally, the solution turned colorless as shown in the inset of Figure 1b.

Polyacrylamide gel electrophoresis of the as-prepared clusters showed two bands. This indicated the presence of two clusters, Ag_7 _and Ag_8 _in the crude solution, which were separated and dissolved in water [34]. The solutions exhibited strong emission. Except for these purified clusters, the excitation and emission are different from the crude clusters without separation (shown in the inset of Figure 1a). The clusters have the molecular formulae, Ag_8_MSA_8 _and Ag_7_MSA_7_, but are described merely as Ag_8 _and Ag_7_. The crude cluster is a 80:20 mixture of Ag_8 _and Ag_7 _which were used for this study. Therefore, we refer to the clusters as Ag_7,8_.

Additional file 1, Figure S1A shows the HRTEM image of the QCs supported on alumina. It was observed that silver QCs were uniformly coated on alumina. They were highly sensitive to the electron beam, and started to fuse and became nanoparticles upon continuous exposure. The lattice fringes with an interplanar spacing of 2.4 Å correspond to Ag (111) (Figure S1B in Additional file 1), indicating the formation of nanoparticles. Corresponding EDAX shows the presence of silver on alumina, as shown in Additional file 1, Figure S1C-E.

**Figure 2:**
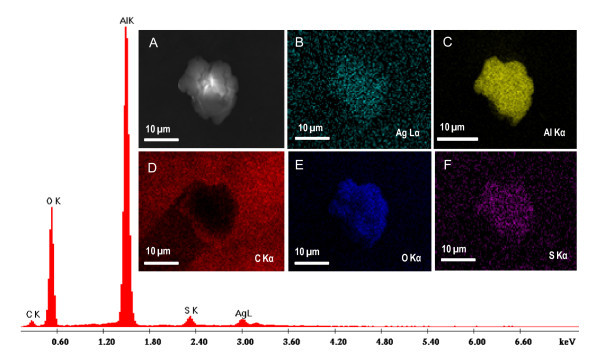
**EDAX spectrum of Al_2_O_3_@Ag_7,8 _along with the quantitative data**. Insets show the SEM image of the supported QCs **(a) **and the elemental maps of an aggregate using Ag Lα **(b)**, Al Kα **(c)**, C Kα **(d)**, O Kα **(e)**, and S Kα **(f)**.

SEM image and EDAX spectrum of Al_2_O_3_@Ag_7,8 _are shown in Figure 2. Elemental maps using appropriate lines are also shown. The spectrum and images confirmed the presence of silver on alumina. It is clear that silver is uniformly coated on alumina. Elemental mapping confirmed the presence of other elements such as sulphur, oxygen, and carbon quantitatively on the alumina matrix. Nearly, 1:1 ratio of Ag:S is observed in the sample. It is confirmed from the data that Ag QCs are protected with MSA.

**Figure 3:**
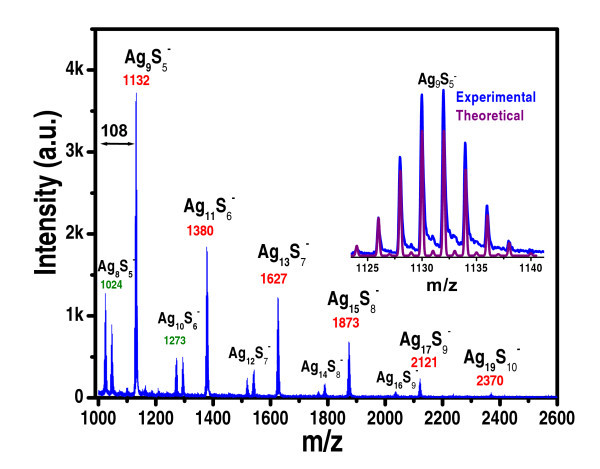
**Negative ion LDI mass spectrum of Ag_7,8 _loaded on alumina**. One of the features is expanded in the inset, along with the theoretical pattern.

Further confirmation of the presence of Ag QCs in the supported material was available from LDI-MS as shown in Figure 3. It gives characteristic features of Ag_n_S_m_^-^. Laser irradiation at 337 nm cleaves AgS-C, bond and Ag_n_S_m _clusters alone are observed in the gas phase. The intact chemical composition of the QC was not observed, as typical of the spectra of thiolated clusters [34,38]. Ag_n_S_m_^- ^species observed in the gas phase are dissociation products as well as gas phase reaction products. Intact clusters, along with the monolayers, are seen only in MALDI-MS and ESI-MS of the free clusters [34]. One of the interesting aspects of silver is its isotope distribution, which helps us to unambiguously assign the ions. To illustrate this, the experimental isotope pattern of one cluster fragment is compared with its theoretical pattern in the inset of Figure 3. It may be noted that the clusters do not fragment upon adsorption on the alumina surface, as properties of the clusters such as luminescence are retained on the oxide surface.

**Figure 4:**
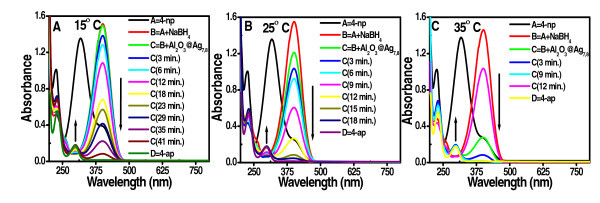
**UV-vis spectra of the reduction of 4-np as a function of time with NaBH_4 _in the presence of supported QCs at 15°C (a), 25°C (b), and 35°C (c)**. Spectrum of 4-ap is given for comparison. Decrease in the concentration of 4-np and corresponding increase in the concentration of 4-ap are marked.

Aqueous solution of 4-np shows characteristic absorption maximum at 317 nm due to the *n *→ π* transition (Figure 4) [40,41]. Upon addition of freshly prepared ice-cold aqueous NaBH_4 _solution, the peak position of 4-np red shifted to 400 nm. This indicates the formation of 4-nitrophenolate ion in alkaline solution. The color of the solution deepened (from pale yellow to deep yellow). Without the addition of clusters, reduction was not observed as seen from the retention of the color. Even for several days, the peak at 400 nm due to 4-nitrophenolate ion remained unaltered.

With the addition of QCs supported on alumina, fading and ultimate leaching of the dark yellow color due to phenolate ions occurred, and brown color of 4-ap appeared. 4-nitrophenolate ion peak at 400 nm got reduced and within 10 min, a new peak around 295 nm appeared due to 4-ap [42,43]. The spectrum of 4-ap was verified with that of a standard sample. Reduction can be visualized with the color change, and it was almost complete which was authenticated by the optical absorbance value of 4-ap. Excess amount of reductant NaBH_4 _was used, and therefore, a pseudo-first-order rate equation may be considered. As a result of adsorption of 4-np and BH_4_^- ^on the cluster surface, electron transfer from donor BH_4_^- ^to the acceptor 4-nitrophenolate ion is facilitated. The reduction was carried out at three different temperatures; 15, 25, and 35°C (Figure 4a,b,c). It was observed that at lower temperatures, the time required was high. Isobestic point observed during the transformation is shown in Additional file 2, Figure S2.

**Figure 5:**
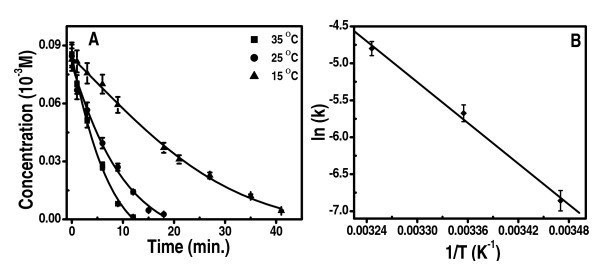
**Kinetics plot for the reduction of 4-np**. **(a) **Variation of 4-np concentration with time in the presence of excess BH_4_^-^. **(b) **A plot of ln(*k*) versus 1/*T *for the reduction of 4-np.

Figure 5A shows the variation of concentration with time of 4-nitrophenolate ion at different temperatures, 15, 25, and 35°C. The rate constants of the reaction (Additional file 3, Table 1) are plotted against 1/T in Figure 5B. Corresponding increase in product concentration is shown in Additional file 4, Figure S3A and B gives the ln(*k*) versus 1/*T *plot for the product formed.

The plot of ln(*k*) versus 1/*T *(Figure 5b) yields a straight line. The activation energy was found to be 55.6 kJ mol^-1 ^at 298 K. The TOF was 1.87 s^-1 ^per cluster. Although the calculated TOF is comparable to that reported for Ag nanoparticles (which is 1-2) [32], we note that certain number of atoms of the clusters are not accessible in the catalytic process as they are used for surface binding. This aspect reduces the available number of surface atoms and increases the TOF per cluster.

Catalysts remain active at the end of the reaction, and these were separated from the product. Again, a fresh batch of 4-np was added to the used catalyst. Fresh reducing agent was not needed till the completion of four cycles, but subsequent cycles required fresh BH_4_^-^. The reaction followed the same kinetics as mentioned above. In this way, four consecutive fresh batches of 4-np were reduced with the same batch of catalyst. Reusability of the same batch of catalyst for the reduction cycles was tested; it remained active for ten cycles. The data for the second, third, fourth, and fifth reduction cycles are shown in Additional file 5, Figure S4. The product obtained was characterized with positive ion ESI-MS as shown in Additional file 6, Figure S5. Formation of 4-ap was confirmed with a peak at *m*/*z *109, and the precursor 4-np peak at *m*/*z *139 also got disappeared. Although the 4-np peak due to protonated 4-np (*m*/*z *110) was not detected (which is the major peak in the pure sample), the molecular ion (*m*/*z *109) is seen in the product. The peak at *m*/*z *109 in the parent compound, 4-np, is due to the loss of NO [44]. This peak in the product spectrum is not due to the presence of unreacted substrate 4-np as its molecular ion signature at *m*/*z *139 disappeared completely.

**Figure 6:**
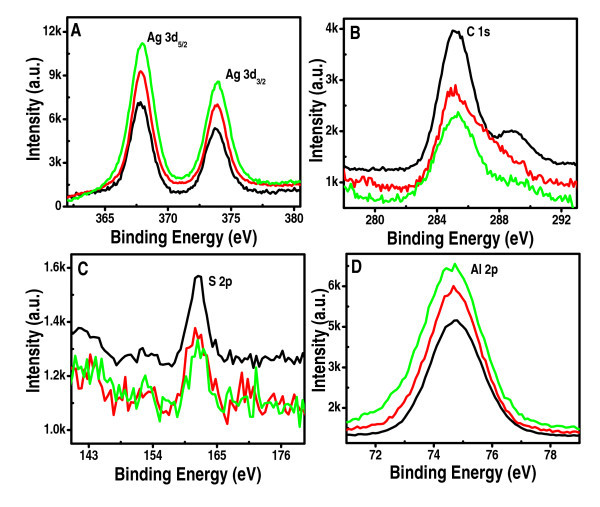
**XPS spectra of supported quantum clusters**. XPS expanded spectra in the Ag 3d **(a)**, C 1s **(b)**, S 2p **(c)**, and Al 2p **(d) **regions of Al_2_O_3_@Ag_7,8 _QCs before (black), after 1st (red) and 3rd cycles (green) of catalysis.

XPS investigation of the catalyst was carried out before and after the reaction. Survey spectra of Al_2_O_3_@Ag_7,8 _before and after the reaction are shown in Additional file 7, Figure S6, and the expanded regions are shown in Figure 6. Spectral shift due to charging was corrected with respect to C 1s at 285.0 eV. Expanded spectra in the Ag 3d region show binding energies of 367.9 and 374.0 eV due to Ag 3d_5/2 _and Ag 3d_3/2_, respectively of Ag (0). S 2p shows a peak at 161.7 eV, Al 2p shows a peak at 74.6 eV, and O 1s appears at 530.8 eV. All the data correspond to the fresh catalyst. The O 1s position indicates hydroxyl groups at the surface, as expected. The C 1s region shows two peaks at 285.0 and 288.3 eV, corresponding to the CH/CH_2 _and -COO^- ^groups. After three cycles, Ag 3d shows peaks at 367.9 and 374.1 eV; corresponding to Ag (0). Al 2p, O 1s, and S 2p did not change significantly. The C 1s region shows a reduction in the peak intensity of the -COO^- ^feature. Reduction in the intensities of sulfur and carbon is noticed. This indicated a slight desorption of the MSA monolayer.

The same experiment was performed with Ag_7,8 _QCs (0.5 mg/0.5 ml) alone (unsupported) in the presence of NaBH_4_, and complete reduction happened within 3 min. The same reduction reaction was repeated with Ag@citrate nanoparticles of 40 nm core diameter supported on alumina. For the supported Ag@citrate nanoparticles (10% loading, 50 mg), the reduction time increased thrice when compared to QCs. It appears that the electron transfer reaction depends upon the surface area of the catalyst, besides the electronic effect. This follows pseudo-first-order kinetics as shown in Additional file 8, Figure S7. The reduction time was reduced with the QCs compared with the nanoparticles.

Other nitro compounds such as 3-na, 4-na, and 3-np were tested with supported QCs (Additional file 9, Figure S8). The peaks at 225 and 358 nm indicate the progress of the reduction of 3-na. The reduction in the peak at 380 nm of 4-na indicates the progress in the reduction with the appearance of the new peaks at 240 and 305 nm. The peaks at 330 and 270 nm reduced with the appearance of the peak at 290 nm during the reduction 3-np.

### Support effect in the catalysis of Ag_7,8_

The same experiment was performed with SiO_2_@Ag_7,8_, TiO_2_@Ag_7,8 _and Fe_2_O_3_@Ag_7,8 _in the presence of NaBH_4_. All the catalyst samples had similar Ag_7,8 _loading (10%). Complete reduction of 4-np happened in 1, 4 and 9 min, and their rate constant values were 1.547 × 10^-1 ^s^-1^, 2.94 × 10^-2 ^s^-1^, and 8.88 × 10^-3 ^s^-1 ^for SiO_2_@Ag_7,8_, TiO_2_@Ag_7,8_, and Fe_2_O_3_@Ag_7,8_, respectively. After the reaction, the catalysts were separated and reused, and the data are shown in Additional file 10, Figure S9. The order of efficiency of the catalyst, in the reduction of 4-np, is SiO_2_@Ag_7,8 _> TiO_2_@Ag_7,8 _> Fe_2_O_3 _@Ag_7,8 _> Al_2_O_3_@Ag_7,8._

## Conclusions

QCs, Ag_7_, and Ag_8 _were supported on various substrates to prepare catalysts, such as Al_2_O_3_@Ag_7,8_, SiO_2_@Ag_7,8_, TiO_2_@Ag_7,8_, and Fe_2_O_3_@Ag_7,8_. Such catalysts show enhanced catalytic activity for the reduction of several nitro compounds. Detailed studies were performed with Al_2_O_3_@Ag_7,8_. The pseudo-first-order rate constant was found to be twice larger than the supported silver of 3.29% loading on an anion exchange resin [27]. The rate constant was found to be 8.23 × 10^-3 ^s^-1^, and the activation energy was 55.6 kJ mol^-1 ^at 298 K. Other nitro aromatics such as 3-np, 3-na, and 4-na were also investigated. The results suggest that the cluster system is a better catalyst for the reactions investigated.

## Abbreviations

ITO: indium tin oxide; MSA: mercaptosuccinic acid; QCs: quantum clusters; SEM: scanning electron microscopy.

## Competing interests

The authors declare that they have no competing interests.

## Authors' contributions

AL conducted the experiments and drafted the manuscript. TUB synthesized the quantum clusters. TP conceived the study, and participated in its design and coordination. All authors read and approved the final manuscript.

## Appendix: Supplementary data

HRTEM-EDAX spectrum and images of Al_2_O_3_@Ag_7,8_, isobestic point in the UV-vis spectra of the reduction of 4-np, increase in concentration of 4-ap during 4-np reduction reaction with corresponding plot of ln(*k*) versus 1/*T*, reusability of the supported Al_2_O_3_@Ag_7,8 _for the reduction of 4-np, positive ion ESI-MS of the product, XPS survey spectra of Al_2_O_3_@Ag_7,8 _after reactions (first and third cycles), UV-vis spectra of the reduction reaction using Ag@citrate nanoparticles with corresponding plot of concentration versus time, variation of the spectral intensities of other nitro aromatics during reduction and UV-vis spectra for the reduction of 4-np with SiO_2_@Ag_7,8_, TiO_2_@Ag_7,8 _and Fe_2_O_3 _@Ag_7,8_. Supplementary data pertaining to with this article can be found, in the online version, in Additional files 1, 2, 3, 4, 5, 6, 7, 8, 9 and 10.

## Supplementary Material

Additional file 1 **Figure S1**. HRTEM image of Al_2_O_3_@Ag_7,8_. Black dots in (A) correspond to Ag QCs which are marked. (B) Lattice-resolved image of fused silver particles obtained after 20 min of electron beam exposure showing the (111) plane of Ag. (C) EADX spectrum of Al_2_O_3_@Ag_7,8 _showing the presence of Ag, corresponding to the elemental map of Al (D) and Ag (E) measured in TEM. Click here for file

Additional file 2 **Figure S2**. Isobestic point in the UV-vis spectra of the reduction of 4-np at 15°C. Minor changes are attributed to the presence of particles of supported clusters in the solution. Click here for file

Additional file 3 **Table 1**. Rate constant for the reduction of 4-np with NaBH_4 _in the presence of Al_2_O_3_@Ag_7,8_ Click here for file

Additional file 4 **Figure S3**. (A) UV-vis spectra of the increase in concentration of 4-ap during the reduction process at 35°C (a), 25°C (b), and 15°C (c). (B) A plot of concentration versus 1/*T *for the formation of 4-ap. Click here for file

Additional file 5 **Figure S4**. Reusability of supported Al_2_O_3_@Ag_7,8 _for the reduction of 4-np, the second cycle (A), the third cycle (B), the fourth cycle (C), and the fifth cycle (D). Click here for file

Additional file 6 **Figure S5**. Positive ion ESI-MS of the product obtained in 50:50 water:methanol mixture, compared with those of pure 4-np and 4-ap. Complete disappearance of the peak of 4-np is noted. Click here for file

Additional file 7 **Figure S6**. XPS survey spectra of Al_2_O_3_@Ag_7,8 _before reaction (black), after the first (red) and the third (green) cycles of reduction reactions. Click here for file

Additional file 8 **Figure S7**. UV-vis spectra for the reduction of 4-np with NaBH_4 _in the presence of supported Ag@citrate nanoparticles. Click here for file

Additional file 9 **Figure S8**. UV-vis spectra for the reduction of 3-na (A), 4-na (B), and 3-np (C) with NaBH_4 _in the presence of Al_2_O_3_@Ag_7,8_. Click here for file

Additional file 10 **Figure S9**. UV-vis spectra for the reduction of 4-np as a function of time, with SiO_2_@Ag_7,8 _(A_1_-A_3_), TiO_2_@Ag_7,8 _(B_1_-B_3_), and Fe_2_O_3 _@Ag_7,8 _(C_1_-C_3_). 1, 2, and 3 refer to the first, second, and third cycles of reduction. Click here for file
