# Supplementary material for: Supported quantum clusters of silver as enhanced catalysts for reduction
Source: Nanoscale Res Lett. 2011 Feb 8;6(1):123. doi: 10.1186/1556-276X-6-123 (PMC3211169; doi:10.1186/1556-276X-6-123)
Supplement: Additional file 1 — Figure S1. HRTEM image of Al2O3@Ag7,8. Black dots in (A) correspond to Ag QCs which are marked. (B) Lattice-resolved image of fused silver particles obtained after 20 min of electron beam exposure showing the (111) plane of Ag. (C) EADX spectrum of Al2O3@Ag7,8 showing the presence of Ag, corresponding to the elemental map of Al (D) and Ag (E) measured in TEM. [file 1556-276X-6-123-S1.DOC]

**Additional file 1, Figure S1**
