# Supplementary material for: Supported quantum clusters of silver as enhanced catalysts for reduction
Source: Nanoscale Res Lett. 2011 Feb 8;6(1):123. doi: 10.1186/1556-276X-6-123 (PMC3211169; doi:10.1186/1556-276X-6-123)
Supplement: Additional file 2 — Figure S2. Isobestic point in the UV-vis spectra of the reduction of 4-np at 15°C. Minor changes are attributed to the presence of particles of supported clusters in the solution. [file 1556-276X-6-123-S2.DOC]

**Additional file 2, Figure S2**
