# Supplementary material for: Supported quantum clusters of silver as enhanced catalysts for reduction
Source: Nanoscale Res Lett. 2011 Feb 8;6(1):123. doi: 10.1186/1556-276X-6-123 (PMC3211169; doi:10.1186/1556-276X-6-123)
Supplement: Additional file 3 — Table 1. Rate constant for the reduction of 4-np with NaBH4 in the presence of Al2O3@Ag7,8 [file 1556-276X-6-123-S3.DOC]

| **Temperature (°C)** | **Rate constants (s^-1^)** |
| --- | --- |
| 15 | 1.05 * 10^-3^ |
| 25 | 3.43 * 10^-3^ |
| 35 | 8.23 * 10^-3^ |

**Additional file 3, Table 1**
