# Supplementary material for: Supported quantum clusters of silver as enhanced catalysts for reduction
Source: Nanoscale Res Lett. 2011 Feb 8;6(1):123. doi: 10.1186/1556-276X-6-123 (PMC3211169; doi:10.1186/1556-276X-6-123)
Supplement: Additional file 4 — Figure S3. (A) UV-vis spectra of the increase in concentration of 4-ap during the reduction process at 35°C (a), 25°C (b), and 15°C (c). (B) A plot of concentration versus 1/T for the formation of 4-ap. [file 1556-276X-6-123-S4.DOC]

**Additional file 4, Figure S3**
