# Supplementary material for: Supported quantum clusters of silver as enhanced catalysts for reduction
Source: Nanoscale Res Lett. 2011 Feb 8;6(1):123. doi: 10.1186/1556-276X-6-123 (PMC3211169; doi:10.1186/1556-276X-6-123)
Supplement: Additional file 5 — Figure S4. Reusability of supported Al2O3@Ag7,8 for the reduction of 4-np, the second cycle (A), the third cycle (B), the fourth cycle (C), and the fifth cycle (D). [file 1556-276X-6-123-S5.DOC]

**Additional file 5, Figure S4**
