# Supplementary material for: Supported quantum clusters of silver as enhanced catalysts for reduction
Source: Nanoscale Res Lett. 2011 Feb 8;6(1):123. doi: 10.1186/1556-276X-6-123 (PMC3211169; doi:10.1186/1556-276X-6-123)
Supplement: Additional file 6 — Figure S5. Positive ion ESI-MS of the product obtained in 50:50 water:methanol mixture, compared with those of pure 4-np and 4-ap. Complete disappearance of the peak of 4-np is noted. [file 1556-276X-6-123-S6.DOC]

**Additional file 6, Figure S5**
