# Supplementary material for: Supported quantum clusters of silver as enhanced catalysts for reduction
Source: Nanoscale Res Lett. 2011 Feb 8;6(1):123. doi: 10.1186/1556-276X-6-123 (PMC3211169; doi:10.1186/1556-276X-6-123)
Supplement: Additional file 7 — Figure S6. XPS survey spectra of Al2O3@Ag7,8 before reaction (black), after the first (red) and the third (green) cycles of reduction reactions. [file 1556-276X-6-123-S7.DOC]

**Additional file 7, Figure S6**
