# Supplementary material for: Supported quantum clusters of silver as enhanced catalysts for reduction
Source: Nanoscale Res Lett. 2011 Feb 8;6(1):123. doi: 10.1186/1556-276X-6-123 (PMC3211169; doi:10.1186/1556-276X-6-123)
Supplement: Additional file 8 — Figure S7. UV-vis spectra for the reduction of 4-np with NaBH4 in the presence of supported Ag@citrate nanoparticles. [file 1556-276X-6-123-S8.DOC]

**Additional 8, Figure S7**
