# Supplementary material for: Supported quantum clusters of silver as enhanced catalysts for reduction
Source: Nanoscale Res Lett. 2011 Feb 8;6(1):123. doi: 10.1186/1556-276X-6-123 (PMC3211169; doi:10.1186/1556-276X-6-123)
Supplement: Additional file 9 — Figure S8. UV-vis spectra for the reduction of 3-na (A), 4-na (B), and 3-np (C) with NaBH4 in the presence of Al2O3@Ag7,8. [file 1556-276X-6-123-S9.DOC]

**Additional file 9, Figure S8**
