# Supplementary material for: Supported quantum clusters of silver as enhanced catalysts for reduction
Source: Nanoscale Res Lett. 2011 Feb 8;6(1):123. doi: 10.1186/1556-276X-6-123 (PMC3211169; doi:10.1186/1556-276X-6-123)
Supplement: Additional file 10 — Figure S9. UV-vis spectra for the reduction of 4-np as a function of time, with SiO2@Ag7,8 (A1-A3), TiO2@Ag7,8 (B1-B3), and Fe2O3 @Ag7,8 (C1-C3). 1, 2, and 3 refer to the first, second, and third cycles of reduction. [file 1556-276X-6-123-S10.DOC]

**Additional file 10, Figure S9**
